# The manatee variational autoencoder model for predicting gene expression alterations caused by transcription factor perturbations

**DOI:** 10.1038/s41598-024-62620-z

**Published:** 2024-05-23

**Authors:** Ying Yang, Lucas Seninge, Ziyuan Wang, Anthony Oro, Joshua M. Stuart, Hongxu Ding

**Affiliations:** 1 Program in Epithelial Biology and Center for Definitive and Curative Medicine, Stanford University, Stanford, CA USA; 2 Department of Biomolecular Engineering and Genomics Institute, University of California, Santa Cruz, Santa Cruz, CA USA; 3 Department of Pharmacy Practice and Science, University of Arizona, Tucson, AZ USA

**Keywords:** Computational models, Machine learning, Computational biology and bioinformatics

## Abstract

We present the Manatee variational autoencoder model to predict transcription factor (TF) perturbation-induced transcriptomes. We demonstrate that the Manatee in silico perturbation analysis recapitulates target transcriptomic phenotypes in diverse cellular lineage transitions. We further propose the Manatee in silico screening analysis for prioritizing TF combinations targeting desired transcriptomic phenotypes.

## Introduction

Predicting cellular transcriptomic responses to perturbations remains challenging. scGen^1^ VAE and Ghahramani et al.^2^ generative adversarial network models exploit the latent space arithmetics for perturbation analyses. Such models take “differential latent space” as a whole to represent the perturbation effect, without providing interpretability to individual latent variables. CPA^3^ autoencoder, together with GEARS^4^ and graphVCI^5^ graph neural network models disentangle perturbation covariates with basal cellular states, thus providing more flexibility for the in silico perturbation analysis. The VEGA^6^ VAE model and the CellOracle^7^ algorithm further increased the perturbation resolution from perturbation covariates which usually conclude an ensemble of functionally-related transcription factors (TFs), to a single TF-level. Specifically, VEGA wires VAE decoders following TF-target connections to correspond latent variables with TFs. However, such a one-layer decoder compromises VEGA generative capacity, therefore impeding the prediction faithfulness. CellOracle propagates perturbation signals in gene regulatory networks to infer lineage trends. However, predicting gene expression values using the CellOracle algorithm remains challenging. More recently, transformer-based large deep learning models, e.g. geneformer^8^, emerged as a new paradigm in single cell analysis. Geneformer transforms single cell expression profiles into a highly-informative latent space, which will further be used for a variety of downstream tasks. Specifically, for in silico perturbation tasks, perturbation effects will be represented further quantified only in the latent space. Thus, geneformer also cannot directly predict perturbation-induced gene expression values. To overcome these limitations, we present the Manatee VAE model to whole transcriptomes in response to perturbations on individual TF modules.

## Results

**Figure 1:**
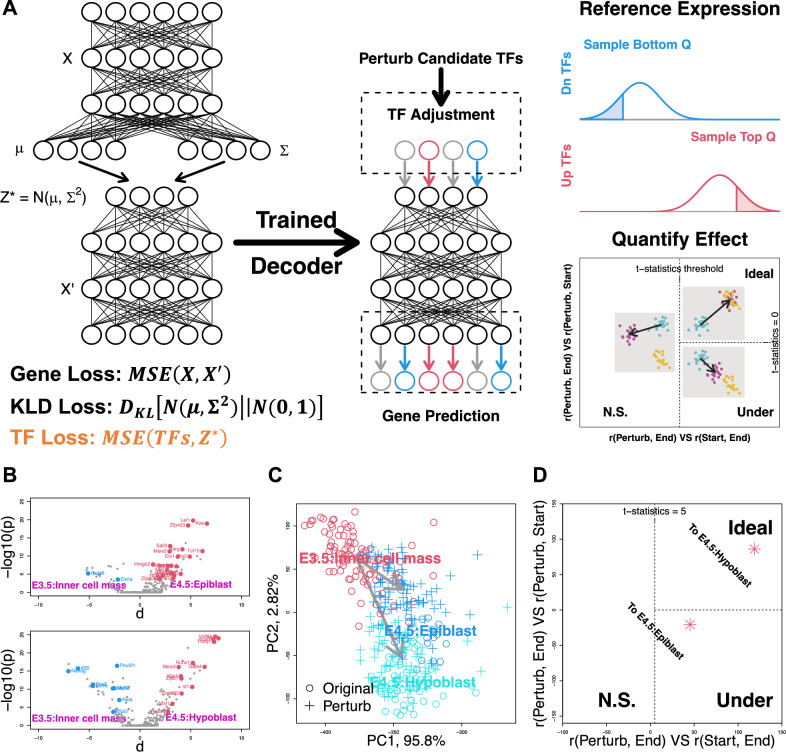
In silico TF perturbation analysis with Manatee. (**A**) Workflow overview. (**B**) Lineage transition TFs highlighted on the TF volcano plot. Up and down-regulated TFs in lineage targets (upper panel hypoblast and lower panel epiblast) were marked as red and blue, respectively. *P* values were calculated using the two-sided U-test. D-values represent average TF expression differences. (**C**) Original (circle) and perturbed (cross) cells projected on the PCA plot. Colors represent cellular identities. Arrows indicate perturbation directions. (**D**) Perturbation effects were projected on the assessment plot.

As shown in Fig. 1A, Manatee is designed to model the generative process from TF expression to whole transcriptomes. To do so, we constrain the Manatee latent space to approximate TF expression by applying an additional loss term during training. The trained decoder network is used to predict gene expression after TF perturbations. Such predictions start with adjusting the latent space. Specifically, for TFs to be up and down-regulated, corresponding latent values are sampled from the user-defined top and bottom Q quantile of the given reference, respectively. Here, we recommend users to take gene expression profiles under real-world biological conditions as the reference, to guarantee a biologically meaningful latent space adjustment. Such adjusted profiles are subsequently flowed through the decoder network for final results. The relative strength among r(*Perturb*, *End*), r(*Start*, *End*) and r(*Perturb*, *Start*) Pearson correlations, which is quantified by t-statistics, is used to evaluate whether a perturbation yields the desired transcriptomic phenotype. Specifically, “Start” refers to the gene expression profile to be perturbed, as the “starting point” of in silico perturbation analysis; “End” refers to the “theoretical” gene expression profile that a correct in silico perturbation analysis should be able to produce; “Perturb” refers to the actual expression profile yielded by in silico perturbation. We next projected t-statistics values on the assessment plot, to distinguish effective (Ideal), compromised (Under) and non-significant (N.S.) perturbations (see “Methods”).

We benchmarked Manatee using the TOME^9^ mouse embryogenesis single cell dataset, which contains 468 cell populations and 113 lineages (see “Methods”). We first evaluated the trained Manatee model, and confirmed that the whole transcriptome could be recovered from TF expression (Figure S1). We then tested whether Manatee in silico perturbation could recapitulate lineage transitions. As an example, we analyzed the lineage bifurcation from inner cell mass to hypoblast and epiblast. We perturbed key TFs documented in TOME (Fig. 1B), and recapitulated both trajectories (Fig. 1C, D). We further analyzed all 113 TOME lineages (Figure S2).

**Figure 2:**
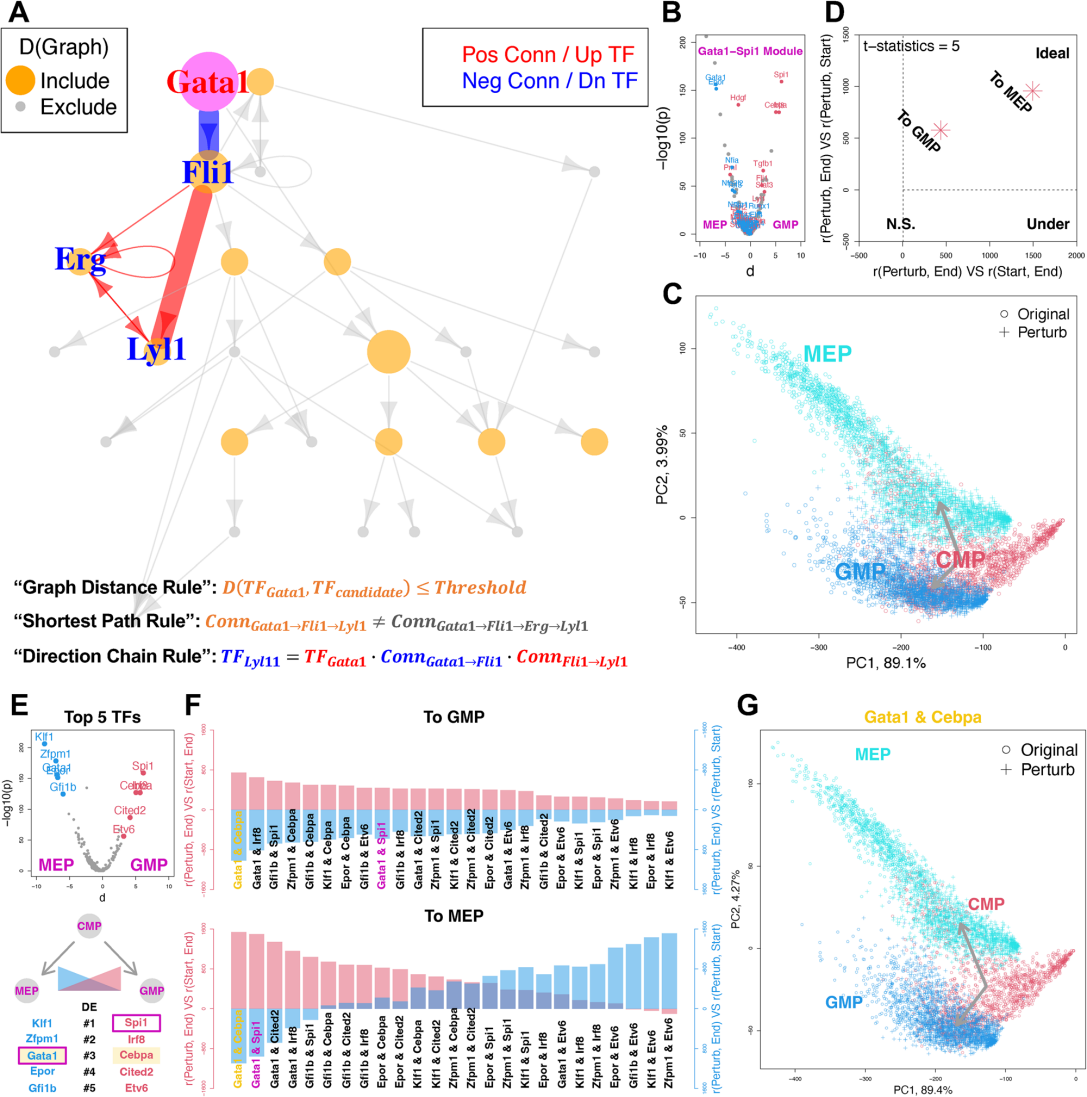
Leveraging TF regulatory information for in silico screening. (**A**) Identify downstream TFs on the TRRUST regulatory network. (**B**) *Gata1*-*Spi1* module targets shown on the TF volcano plot. Up and down-regulated targets between GMP and MEP were marked as red and blue, respectively. P-values and d-values were calculated as in Fig. 1. (**C, D**) PCA and assessment plots showing perturbation effects, as in Fig. 1. (**E**) Top 5 highly expressed TFs in MEP and GMP. (**F**) TF combinations ranked by perturbation strengths. (**G**) PCA plot for the top-ranked *Gata1*-*Cebpa* combination.

Real-world biological experiments are usually designed to investigate a specific master regulatory TF module. Its downstream TFs, which are required for an effective Manatee prediction, are in general not provided. To identify such downstream TFs, we referred to the TRRUST^10^ TF regulatory target database. As shown in Fig. 2A, target TFs downstream of the regulatory module are determined following three rules. First, only TFs within a certain graph distance radius are included (“Graph Distance Rule”). Second, only the shortest path to a target is used to calculate the regulatory direction (“Shortest Path Rule”). Third, the direction is calculated with a “Exclusive-NOR gate chain” (up-regulated TF/positive connection as 1, and vice versa) along the regulatory path (“Direction Chain Rule”). These identified TFs, together with TFs in the regulatory module, will be in silico perturbed simultaneously for the reliable Manatee analysis. We benchmarked such a TRRUST-powered Manatee analysis using the common myeloid progenitor (CMP) to granulocyte/macrophage progenitor (GMP) VS megakaryocyte/erythrocyte progenitor (MEP) lineage bifurcation, which is driven by the *Gata1*-*Spi1* module^11^ (see “Methods”). We trained and evaluated the Manatee model (Figure S3), identified target TFs (Fig. 2B), and recapitulated both trajectories (Fig. 2C, D). We further confirmed that Manatee in silico perturbation is biologically valid, by recovering cell type-specific marker genes (Figure S4).

We leveraged Manatee for the in silico screening of perturbations that could yield the target transcriptomic phenotype. As a proof-of-concept, we screened alternative TF duos driving the above hematopoiesis process. Without losing generality, we screened all 25 combinations of the top 5 highly expressed TFs within MEP and GMP (Fig. 2E), and ranked them based on perturbation effectiveness (Figs. 2F and S5). We prioritized the *Gata1*-*Cebpa* combination, which could effectively guide lineage transitions (Fig. 2G) as well as recapitulate marker gene expression (Figure S6). Noticeably, *Cebpa* has been experimentally validated as a master regulator controlling the identity of GMP^11^.

## Discussion

Manatee shares architectural similarity with the semantic autoencoder^12^. Both models present a “semantically meaningful” latent space, which is achieved by an additional latent space loss term. The latent space of the conventional VAE, in contrast, is in general less interpretable. By this means, Manatee presents a new group of VAE architecture. The critical difference between Manatee and semantic autoencoders is the generative capacity. As a VAE, Manatee is capable of producing biologically meaningful transcriptomic predictions from in silico perturbed TF latent variables.

We acknowledge that the current Manatee architecture cannot properly handle dropouts. As a result, reconstructed transcriptomes (X’) and reparameterized TF profiles (Z*) tend to be “imputed” (Figures S1 and S3). Such a data imputation compromises the r(X, X’) and r(Z, Z*) Pearson correlation, especially for cells with a smaller transcriptome complexity. To model dropouts, a future direction would be to use a scVI-like decoder^13^.

We also acknowledge that the current Manatee design is less likely to represent actual gene regulatory interactions. That being said, TF perturbation effects cannot be automatically disseminated to downstream target TFs. Thus, besides candidates, their downstream TFs should also be adjusted for reliable predictions. We tackled such a problem by introducing the TRRUST network to identify the entire TF regulatory module. Besides perturbing TFs, other common biological use cases are to perturb signaling proteins or even the upstream pathways. To expand the scope of Manatee, one future direction would be to include, e.g. the STRING^14^ functional protein–protein association network in the Manatee workflow. We also propose the multi-layer regulatory network decoder as an alternative architecture for addressing the limitation. We speculate that such a design would balance the model interpretability and generative capability.

We find properly adjusting the Manatee latent space to be challenging. This is because, without prior biological knowledge, it is extremely difficult to precisely set latent variables. As a result, even with proper TF sets and regulatory directions, artifacts could still happen. As shown in Figure S7, underestimated latent values might result in “under” perturbations. In such scenarios, cells are moving towards the right direction but still distant from the target transcriptomic phenotype. On the other hand, overestimating TF profiles might lead to over-perturbed cells. Such cells usually become less similar to the target therefore fall into the ineffective “N.S.” group. We also find high quality TF sets to be crucial for Manatee predictions. Reducing the number of candidate TFs might cause compromised perturbations (Figure S7), while reversing TF regulatory directions or providing biologically meaningless random TF sets could yield “N.S.” perturbations (Figure S8). To fully appreciate the power of Manatee, we propose two potential future directions. To make accurate latent variable adjustments, it might be necessary to establish perturbation dosage-TF expression alteration correspondences. To determine proper perturbation candidates, comprehensive, accurate and biological context-specific TF annotations are needed.

We observe that Manatee has the capability to encode diverse biological states all together, e.g. TOME cell states and trajectories. We also observe that disrupting TF correlations by randomly shuffling TF profiles compromises transcriptome reconstruction (Figures S1 and S3). We thus conclude the successful encoding of biological information by Manatee, and attribute the encoding potential to the neural network depth and width. We further speculate additional cell populations could be included, for training a model capable of representing all major biological processes, both in vivo and in vitro, related to a certain species. We consider establishing such a model to be a future direction.

We anticipate a broad application of Manatee, in particular for diverse in silico screening use cases. For instance, Manatee could be used to screen for differentiation strategies that push stem cells to certain cell types, as well as therapeutics that reverse diseased transcriptomic signatures. We also anticipate Manatee to identify perturbations that cause “aberrant” transcriptomic phenotypes, e.g. drug side effects. We speculate that Manatee is unlikely to represent biological states that have never been seen during training. That being said, Manatee might yield artifacts when dealing with “aberrant” transcriptomes. Thus the above-mentioned “comprehensive model” would be of great value. Meanwhile, one could incorporate novel biological states into existing models by applying incremental learning techniques. We thus provide a trained TOME mouse embryogenesis Manatee model for further development.

## Methods

### Data preparation

TOME comprehensively described mouse gastrulation and organogenesis using > 1,600,000 expression profiles, 19 developmental stages ranging from E3.5 to E13.5, 468 cell populations and 113 lineages. These normalized expression profiles and cell type/lineage annotations were stored in Seurat objects, and are available at the TOME data portal. To create gene-by-cell expression matrices for training and benchmarking Manatee, we downloaded the full TOME dataset, then filtered genes whose expression rates are less than 10% in each cell population. We then took the union of the remaining genes as the final gene list. Such a selection process aims at preserving genes that are uniquely expressed in small cell populations. TOME lineage transition-specific TF sets were collected from the Table S7 and S9 of the original paper. We organize the union of lineage transition-specific TFs into the final TF list. We randomly sampled 200 and 100 cells per cell population to build train and test datasets, respectively.

Dataset GSE72857 described the CMP to GMP VS MEP mouse hematopoiesis lineage bifurcation. We directly downloaded normalized expression profiles, as well as the metadata spreadsheet, from the GSE72857 repository. To better represent in vivo developmental processes, we took single cells annotated as “unsorted myeloid” for in silico perturbation analysis (GSE72857 also contains expression profiles from knock-out experiments). To create the gene-by-cell expression matrix for Manatee-based in silico perturbation and screening, we combined all unsorted myeloid cells and filtered genes whose expression rates are less than 10%. We didn’t do (1) per-cell type gene filtering, considering the relatively balanced proportions between CMP, GMP and MEP; (2) cell down-sampling, considering the relatively small number of unsorted myeloid cells (~ 3000). We took the union of TOME TFs as above-described, as well as TRRUST TFs as the final TF list for GSE72857.

### The manatee architecture

Manatee is adapted from a VAE^15^ architecture designed to model the generative process from TF profiles to corresponding transcriptomes. In order to do so, (1) both the encoder and decoder neural networks consist of the same number of fully connected layers (three for this study), each with the same number of nodes as the number of genes (17,559 for TOME, 4342 for GSE72857); (2) the latent space contains the same number of latent variables as the number of TFs (693 for TOME, 296 for GSE72857); and (3) the following loss function is optimized during training:

$$L = \left( {1 - \alpha } \right) \times \{ \left( {1 - \beta } \right) \times L_{R} + \beta \times D_{KL} \} + \alpha \times L_{r}$$

where *L*_*R*_ and *D*_*KL*_ represent reconstruction loss and Kullback–Leibler Divergence against the *N(0, ****1)*** normal distribution respectively, as the two regular VAE loss terms. The additional *L*_*r*_ term represents the TF reconstruction loss, which is the mean square error (MSE) between reparameterized latent variables (***Z***) and TF expression (***TF***):

$$L_{r} \equiv MSE\left( {Z, TF} \right)$$

Coefficient α balance regular VAE loss terms *L*_*R*_ and *D*_*KL*_, while coefficient β balance the regular VAE loss and the added TF reconstruction loss term *L*_*r*_. We considered α and β to be the two major hyper-parameter to be tuned when training the Manatee model. Our hyper-parameter tuning suggested {α = 0.8, β = 5e−05} to be an optimal choice, which was used for training both TOME and GSE72857 models. We further confirmed that models trained with this choice can properly encode biology, by recapitulating 1) TF expression profiles after reparametrization, and 2) gene expression profiles using the decoder, as shown in Figure S1 and S3.

### Manatee latent space adjustment

Manatee in silico perturbation starts with adjusting latent values corresponding to candidate TFs. In order to produce a legitimate latent space, TF values will be sampled from the reference matrix, which tracks expression patterns in actual biological settings. In this study, we used the full expression matrices as references. For TFs to be up and down-regulated, corresponding values were sampled with replacement from the top and bottom Q quantile of the reference. In this study, Q was set as 1%. We adopted such an “extreme” Q to make sure only representative values are used. Our rationales being that (1) we observed that TF value distributions between original and target phenotypes are in general close to each other, and (2) a specific cell population only accounts for a very small proportion of the entire TOME collection. For TFs not marked as candidates, we kept their original expression values.

### Determining candidate TFs and regulatory directions from the TRRUST network

We leveraged the TRRUST transcriptional regulatory network to identify the full TF set downstream of a specific TF module. We first filtered TRRUST by only keeping TF-TF interactions with clear direction annotation. We further removed duplicated edges and loops within TRRUST network. The cleaned-up TRRUST network was then used for determining target TFs and regulatory directions. As mentioned in the main text, our TRRUST analysis follows three rules, including “Graph Distance Rule”, “Shortest Path Rule” and “Direction Chain Rule”. We set the graph distance threshold as 6 for mouse hematopoiesis analyses. Even constrained by the “Shortest Path Rule”, multiple paths might be identified between two TFs. For such cases, we took the average regulatory directions calculated from the “Direction Chain Rule” as final direction assignments. The rationale being that, if both positive and negative regulations exist, their regulatory effect should cancel out and the direction with more known paths should dominate. We took TFs with positive and negative “average directions” as up and down-regulated targets. The above TRRUST analyses were performed using R package igraph version 1.2.4.1 under R version 3.5.0.

### Supplementary Information

Supplementary Information.

## Data Availability

The datasets generated and/or analyzed during the current study are available in the TOME repository, http://tome.gs.washington.edu/; the Gene Expression Omnibus repository under accession number GSE72857, https://www.ncbi.nlm.nih.gov/geo/query/acc.cgi?acc=GSE72857; the TRRUST repository, https://www.grnpedia.org/trrust/.

## Code availability

The Manatee algorithm is available at https://github.com/hd2326/Manatee.
