# Supplementary material for: The manatee variational autoencoder model for predicting gene expression alterations caused by transcription factor perturbations
Source: Sci Rep. 2024 May 23;14:11794. doi: 10.1038/s41598-024-62620-z (PMC11116378; doi:10.1038/s41598-024-62620-z)
Supplement: Supplementary file 1 — Supplementary Information. [file 41598_2024_62620_MOESM1_ESM.pdf]

Manatee: a Variational Autoencoder Model for Predicting Gene Expression Alterations Caused by Transcription Factor Perturbations

Supplementary Information

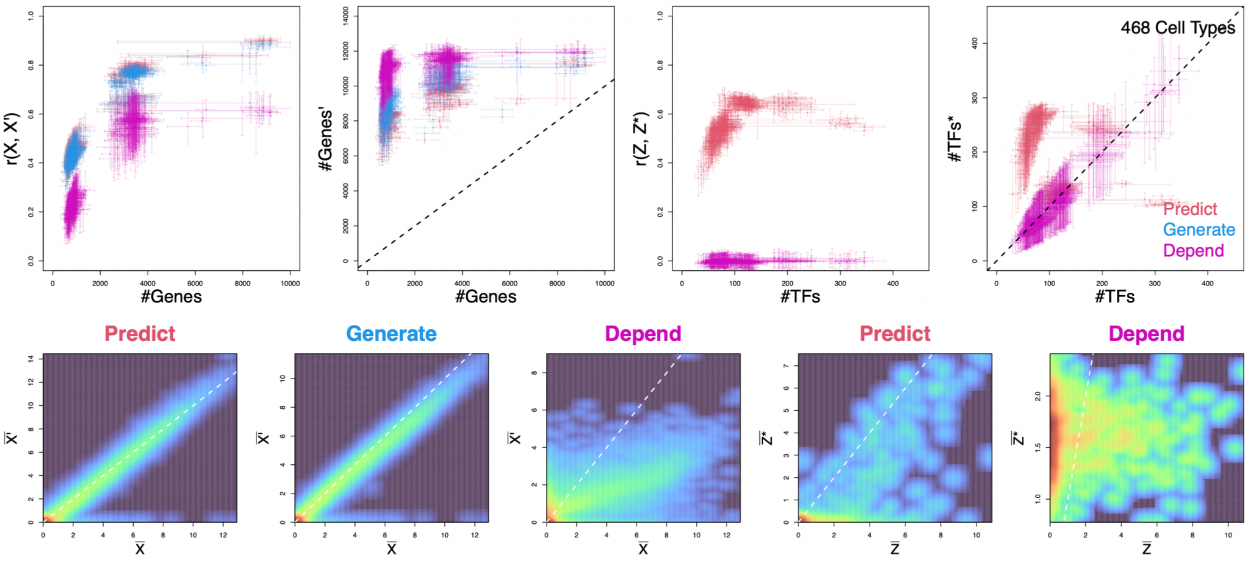

r(X, X')
#Genes
#Genes'
r(Z, Z*)
#TFs
#TFs*
468 Cell Types
Predict
Generate
Depend
Predict
Generate
Depend
Predict
Depend

Figure S1. TOME model benchmarking. We examined whether the TOME model is able to capture biological information. Specifically, we benchmarked the two modes of Manatee. The "Predict" mode encodes original transcriptomes (X), reparametrizes the latent space as decoder inputs Z*, and reconstructs transcriptomes (X'). The "Generate" mode, on the other hand, directly decodes X' from Z*. We assessed the performance of Manatee with the r(X, X') Pearson correlation. We noticed that cells with >3,000 #Genes achieved a ~0.8 r(X, X'), while cells with smaller transcriptomes produced compromised r(X, X'). We explained the trend as an artifact caused by dropout imputation: regardless of #Genes, #Genes' in X' tends to be similar. We further quantified r(Z, Z*) between original (Z) and Z* TF profiles, as well as compared number of TFs detected in Z (#TFs) and Z* (#TFs*) in the "Predict" group, and got similar conclusions as for the gene-level analysis. We then tested if the TOME model is able to recapitulate transcriptomes with de-correlated TF profiles. Such a "Depend" group used the Manatee "Generate" mode by taking randomly shuffled TF profiles as latent space inputs Z*. We observed impeded transcriptome reconstruction with lower r(X, X'). We thus concluded that Manatee takes advantage of biological associations among TFs to reconstruct transcriptomes, further confirming the encoding of biological information. We finally provided smooth scatter visualizations (color palette represents spatial density of genes or TFs) of average X VS X' in "Predict", "Generate" and "Depend" groups, as well as average Z VS Z* in "Predict" and "Depend" groups. Without losing generality, we chose the cell type "E3.5:Inner cell mass" as an example for visualization.

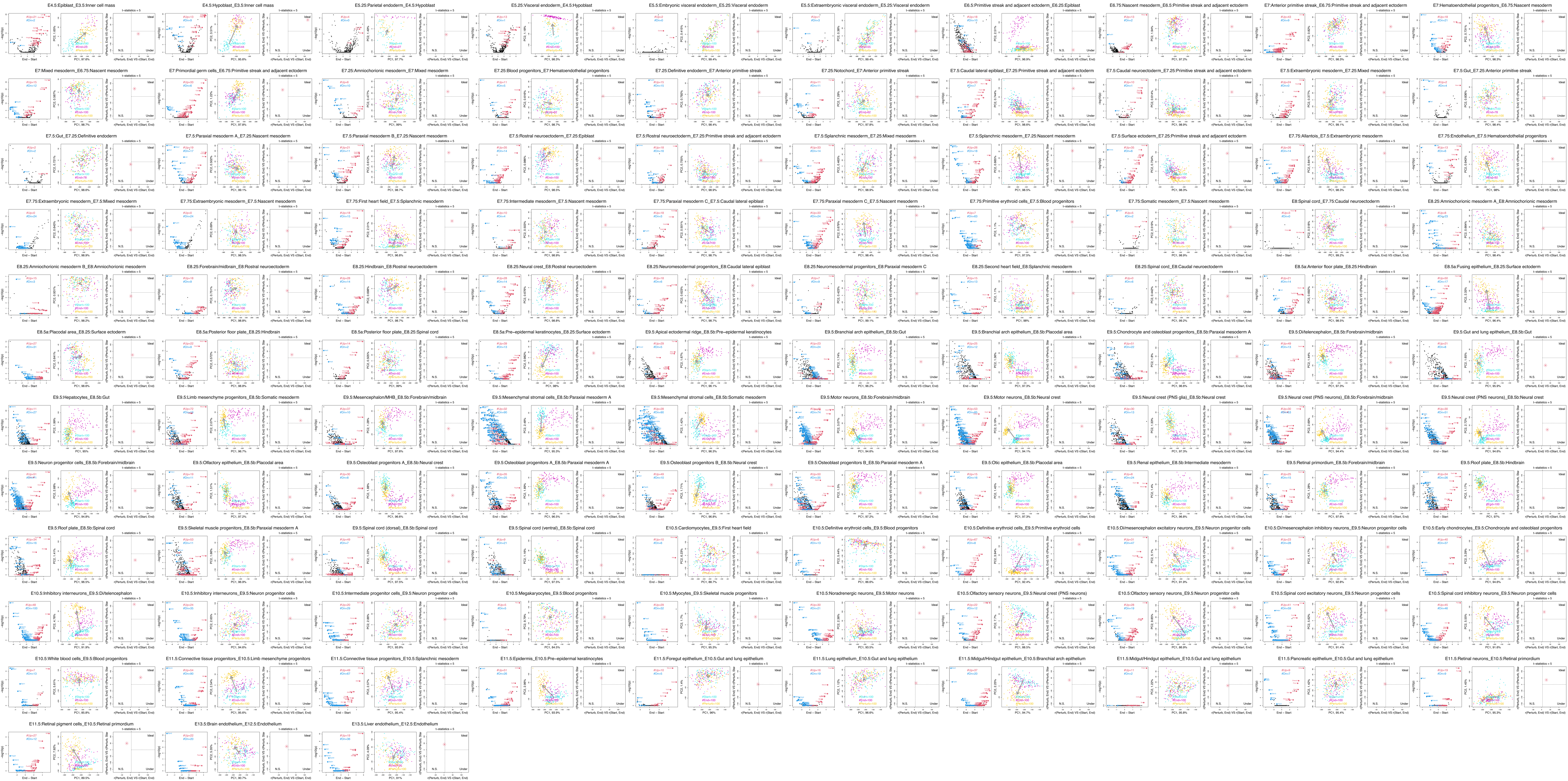
E4.5:Epiblast_E3.5:Inner cell mass
E4.5:Hypoblast_E3.5:Inner cell mass
E5.25:Parietal endoderm_E4.5:Hypoblast
E5.25:Visceral endoderm_E4.5:Hypoblast
E5.5:Embryonic visceral endoderm_E5.25:Visceral endoderm
E5.5:Extraembryonic visceral endoderm_E5.25:Visceral endoderm
E6.5:Primitive streak and adjacent ectoderm_E6.25:Epiblast
E6.75:Nascent mesoderm_E6.5:Primitive streak and adjacent ectoderm
E7:Anterior primitive streak_E6.75:Primitive streak and adjacent ectoderm
E7:Hematoendothelial progenitors_E6.75:Nascent mesoderm
E7:Mixed mesoderm_E6.75:Nascent mesoderm
E7:Primordial germ cells_E6.75:Primitive streak and adjacent ectoderm
E7.25:Amniochorionic mesoderm_E7:Mixed mesoderm
E7.25:Blood progenitors_E7:Hematoendothelial progenitors
E7.25:Definitive endoderm_E7:Anterior primitive streak
E7.25:Notochord_E7:Anterior primitive streak
E7.5:Caudal lateral epiblast_E7.25:Primitive streak and adjacent ectoderm
E7.5:Caudal neuroectoderm_E7.25:Primitive streak and adjacent ectoderm
E7.5:Extraembryonic mesoderm_E7.25:Mixed mesoderm
E7.5:Gut_E7.25:Anterior primitive streak
E7.5:Gut_E7.25:Definitive endoderm
E7.5:Paraxial mesoderm A_E7.25:Nascent mesoderm
E7.5:Paraxial mesoderm B_E7.25:Nascent mesoderm
E7.5:Rostral neuroectoderm_E7.25:Epiblast
E7.5:Rostral neuroectoderm_E7.25:Primitive streak and adjacent ectoderm
E7.5:Splanchnic mesoderm_E7.25:Mixed mesoderm
E7.5:Splanchnic mesoderm_E7.25:Nascent mesoderm
E7.5:Surface ectoderm_E7.25:Primitive streak and adjacent ectoderm
E7.75:Allantois_E7.5:Extraembryonic mesoderm
E7.75:Endothelium_E7.5:Hematoendothelial progenitors
E7.75:Extraembryonic mesoderm_E7.5:Mixed mesoderm
E7.75:Extraembryonic mesoderm_E7.5:Nascent mesoderm
E7.75:First heart field_E7.5:Splanchnic mesoderm
E7.75:Intermediate mesoderm_E7.5:Nascent mesoderm
E7.75:Paraxial mesoderm C_E7.5:Caudal lateral epiblast
E7.75:Paraxial mesoderm C_E7.5:Nascent mesoderm
E7.75:Primitive erythroid cells_E7.5:Blood progenitors
E7.75:Somatic mesoderm_E7.5:Nascent mesoderm
E8:Spinal cord_E7.75:Caudal neuroectoderm
E8.25:Amniochorionic mesoderm A_E8:Amniochorionic mesoderm
E8.25:Amniochorionic mesoderm B_E8:Amniochorionic mesoderm
E8.25:Forebrain/midbrain_E8:Rostral neuroectoderm
E8.25:Hindbrain_E8:Rostral neuroectoderm
E8.25:Neural crest_E8:Rostral neuroectoderm
E8.25:Neuromesodermal progenitors_E8:Caudal lateral epiblast
E8.25:Neuromesodermal progenitors_E8:Paraxial mesoderm C
E8.25:Second heart field_E8:Splanchnic mesoderm
E8.25:Spinal cord_E8:Caudal neuroectoderm
E8.5a:Anterior floor plate_E8.25:Hindbrain
E8.5a:Fusing epithelium_E8.25:Surface ectoderm
E8.5a:Placodal area_E8.25:Surface ectoderm
E8.5a:Posterior floor plate_E8.25:Hindbrain
E8.5a:Posterior floor plate_E8.25:Spinal cord
E8.5a:Pre−epidermal keratinocytes_E8.25:Surface ectoderm
E9.5:Apical ectodermal ridge_E8.5b:Pre−epidermal keratinocytes
E9.5:Branchial arch epithelium_E8.5b:Gut
E9.5:Branchial arch epithelium_E8.5b:Placodal area
E9.5:Chondrocyte and osteoblast progenitors_E8.5b:Paraxial mesoderm A
E9.5:Di/telencephalon_E8.5b:Forebrain/midbrain
E9.5:Gut and lung epithelium_E8.5b:Gut
E9.5:Hepatocytes_E8.5b:Gut
E9.5:Limb mesenchyme progenitors_E8.5b:Somatic mesoderm
E9.5:Mesencephalon/MHB_E8.5b:Forebrain/midbrain
E9.5:Mesenchymal stromal cells_E8.5b:Paraxial mesoderm A
E9.5:Mesenchymal stromal cells_E8.5b:Somatic mesoderm
E9.5:Motor neurons_E8.5b:Forebrain/midbrain
E9.5:Motor neurons_E8.5b:Neural crest
E9.5:Neural crest (PNS glia)_E8.5b:Neural crest
E9.5:Neural crest (PNS neurons)_E8.5b:Forebrain/midbrain
E9.5:Neural crest (PNS neurons)_E8.5b:Neural crest
E9.5:Neuron progenitor cells_E8.5b:Forebrain/midbrain
E9.5:Olfactory epithelium_E8.5b:Placodal area
E9.5:Osteoblast progenitors A_E8.5b:Neural crest
E9.5:Osteoblast progenitors A_E8.5b:Paraxial mesoderm A
E9.5:Osteoblast progenitors B_E8.5b:Neural crest
E9.5:Osteoblast progenitors B_E8.5b:Paraxial mesoderm A
E9.5:Otic epithelium_E8.5b:Placodal area
E9.5:Renal epithelium_E8.5b:Intermediate mesoderm
E9.5:Retinal primordium_E8.5b:Forebrain/midbrain
E9.5:Roof plate_E8.5b:Hindbrain
E9.5:Roof plate_E8.5b:Spinal cord
E9.5:Skeletal muscle progenitors_E8.5b:Paraxial mesoderm A
E9.5:Spinal cord (dorsal)_E8.5b:Spinal cord
E9.5:Spinal cord (ventral)_E8.5b:Spinal cord
E10.5:Cardiomyocytes_E9.5:First heart field
E10.5:Definitive erythroid cells_E9.5:Blood progenitors
E10.5:Definitive erythroid cells_E9.5:Primitive erythroid cells
E10.5:Di/mesencephalon excitatory neurons_E9.5:Neuron progenitor cells
E10.5:Di/mesencephalon inhibitory neurons_E9.5:Neuron progenitor cells
E10.5:Early chondrocytes_E9.5:Chondrocyte and osteoblast progenitors
E10.5:Inhibitory interneurons_E9.5:Di/telencephalon
E10.5:Inhibitory interneurons_E9.5:Neuron progenitor cells
E10.5:Intermediate progenitor cells_E9.5:Neuron progenitor cells
E10.5:Megakaryocytes_E9.5:Blood progenitors
E10.5:Myocytes_E9.5:Skeletal muscle progenitors
E10.5:Noradrenergic neurons_E9.5:Motor neurons
E10.5:Olfactory sensory neurons_E9.5:Neural crest (PNS neurons)
E10.5:Olfactory sensory neurons_E9.5:Neuron progenitor cells
E10.5:Spinal cord excitatory neurons_E9.5:Neuron progenitor cells
E10.5:Spinal cord inhibitory neurons_E9.5:Neuron progenitor cells
E10.5:White blood cells_E9.5:Blood progenitors
E11.5:Connective tissue progenitors_E10.5:Limb mesenchyme progenitors
E11.5:Connective tissue progenitors_E10.5:Splanchnic mesoderm
E11.5:Epidermis_E10.5:Pre−epidermal keratinocytes
E11.5:Foregut epithelium_E10.5:Gut and lung epithelium
E11.5:Lung epithelium_E10.5:Gut and lung epithelium
E11.5:Midgut/Hindgut epithelium_E10.5:Branchial arch epithelium
E11.5:Midgut/Hindgut epithelium_E10.5:Gut and lung epithelium
E11.5:Pancreatic epithelium_E10.5:Gut and lung epithelium
E11.5:Retinal neurons_E10.5:Retinal primordium
E11.5:Retinal pigment cells_E10.5:Retinal primordium
E13.5:Brain endothelium_E12.5:Endothelium
E13.5:Liver endothelium_E12.5:Endothelium

Figure S2. Manatee *in silico* perturbation of all TOME lineages. We performed Manatee *in silico* perturbation analysis on all the 113 TOME lineages. Specifically, we highlighted lineage transition TFs on the TF volcano plot. Up and down-regulated TFs in lineage targets were marked as red and blue, respectively. P-values were calculated using the two-sided U-test. D-values represent average TF expression differences. We further visualized the perturbation effect using the PCA plot. Cells were color-coded based on their groups, including original (Start, yellow), target (End, pink) and perturbed (Perturb, orange) cell groups. Arrows indicate predicted perturbation directions. We further quantified perturbation effects using the assessment plot.

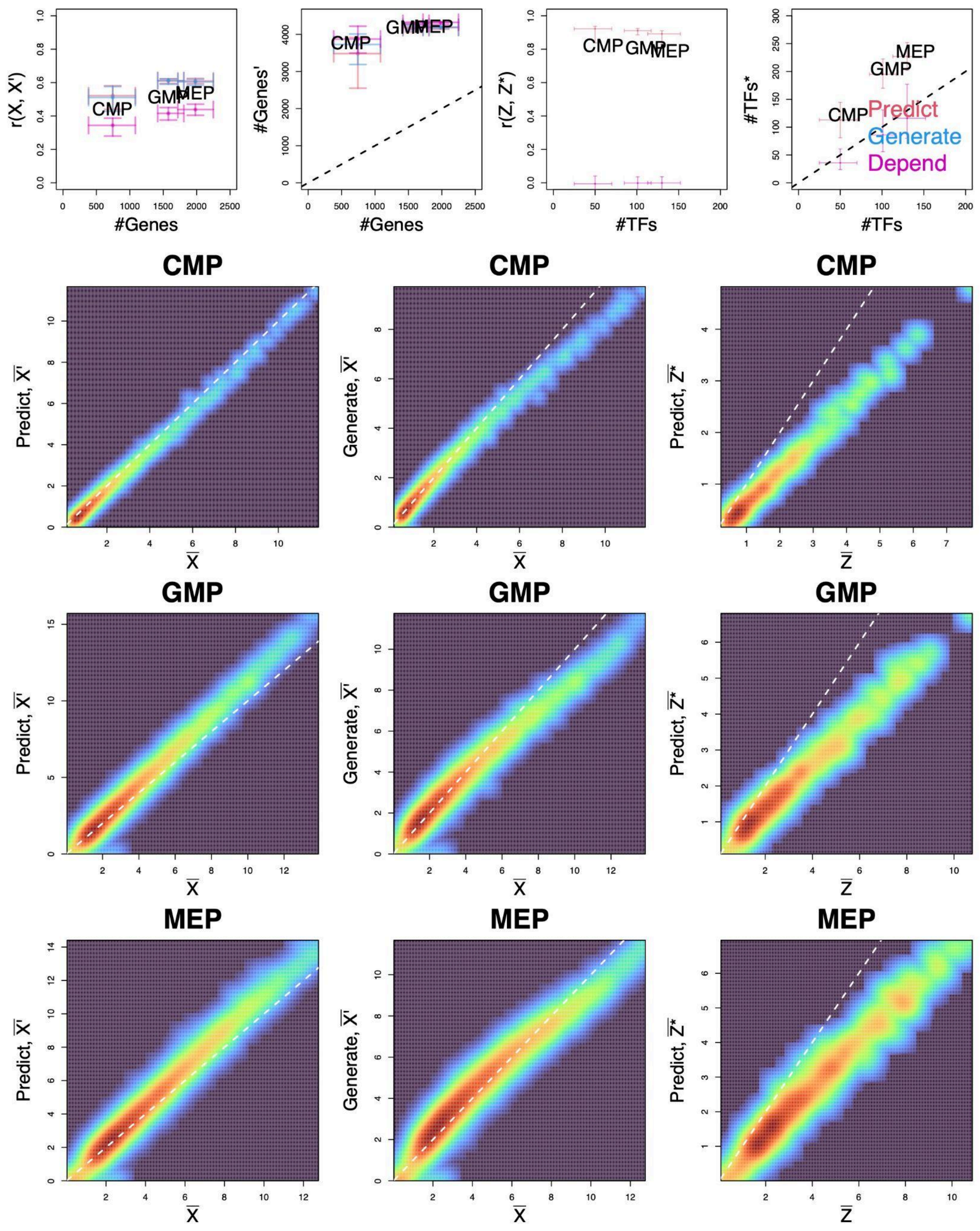
r(X, X')
#Genes
CMP
GMP
MEP
#Genes'
#Genes
r(Z, Z*)
#TFs
#TFs*
#TFs
Predict
Generate
Depend
CMP
GMP
MEP
Predict, $\overline{X'}$
Generate, $\overline{X'}$
Predict, $\overline{Z^*}$
$\overline{X}$
$\overline{Z}$

Figure S3. Mouse hematopoiesis model benchmarking. We examined whether the mouse hematopoiesis model is able to capture biological information. Same as TOME, we benchmarked both “Predict” and “Generate” modes, and confirmed the importance of TF-TF associations in transcriptome reconstruction. We also presented smooth scatter plots (color palette represents spatial density of genes or TFs) of per-cell type average X VS X’ and average Z VS Z* to visualize the performance of reconstruction and reparameterization, respectively.

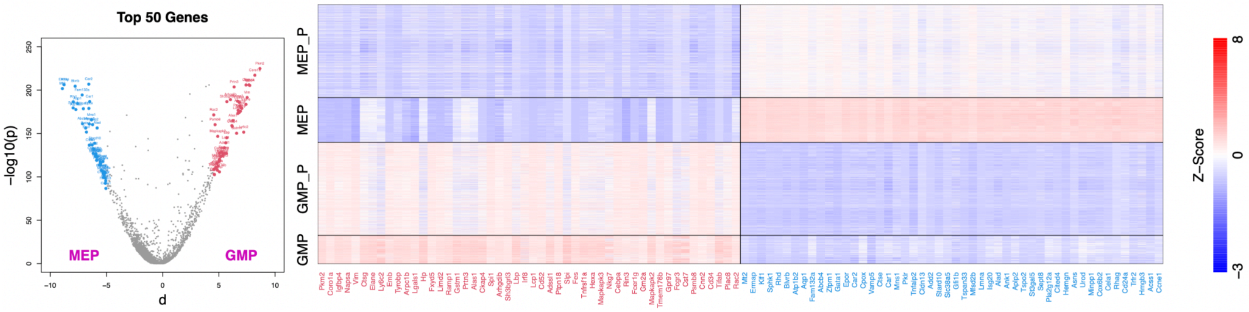
Top 50 Genes
−log10(p)
d
MEP
GMP
MEP_P
MEP
GMP_P
GMP
Z−Score
8
0
−3

Figure S4. Marker expression in *Gata1-Spi1* module-induced transcriptomes. We examined whether key markers could be recapitulated by Manatee predictions. We defined GMP and MEP markers as the top 50 highly expressed genes. We highlighted such genes on the gene volcano plot. Markers for GMP and MEP were marked as red and blue, respectively. P-values were calculated using the two-sided U-test. D-values represent average gene expression differences. We further visualized the behavior of such markers within original GMP and MEP cells, as well as predicted GMP (GMP_P) and MEP (MEP_P) cells using the normalized expression heatmap. Gene-wise z-scores were used to quantify normalized expression profiles. We found the recapitulation of marker expression by Manatee.

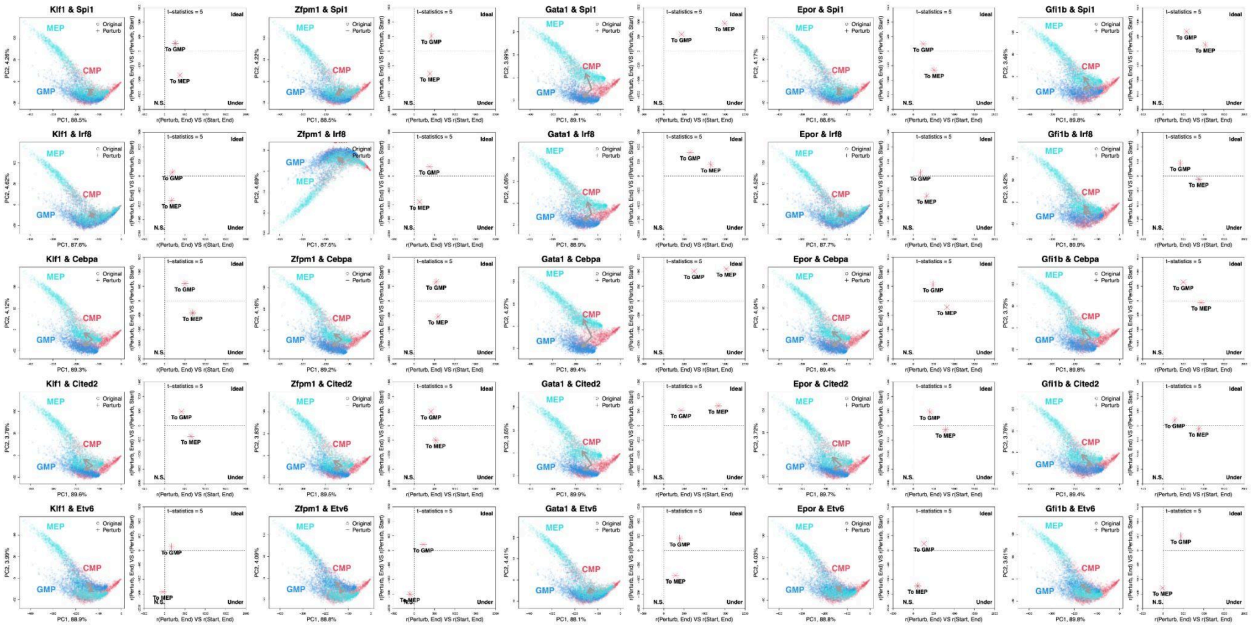

Klf1 & Spi1
Zfpm1 & Spi1
Gata1 & Spi1
Epor & Spi1
Gfi1b & Spi1
Klf1 & Irf8
Zfpm1 & Irf8
Gata1 & Irf8
Epor & Irf8
Gfi1b & Irf8
Klf1 & Cebpa
Zfpm1 & Cebpa
Gata1 & Cebpa
Epor & Cebpa
Gfi1b & Cebpa
Klf1 & Cited2
Zfpm1 & Cited2
Gata1 & Cited2
Epor & Cited2
Gfi1b & Cited2
Klf1 & Etv6
Zfpm1 & Etv6
Gata1 & Etv6
Epor & Etv6
Gfi1b & Etv6
MEP
CMP
GMP
Original
Perturb
t-statistics = 5
Ideal
N.S.
Under
To GMP
To MEP
r(Perturb, End) VS r(Start, End)
r(Perturb, End) VS r(Perturb, Start)

Figure S5. Manatee *in silico* perturbation of all screened TF combinations. We performed Manatee *in silico* perturbation analysis on all the TF combinations to be screened. We visualized the perturbation effect using the PCA plot. Circles and crosses mark original and perturbed cells, respectively. Cells were color-coded based on their groups, including CMP (red), GMP (blue) and MEP (cyan). Arrows indicate predicted perturbation directions. We further quantified perturbation effects using the assessment plot.

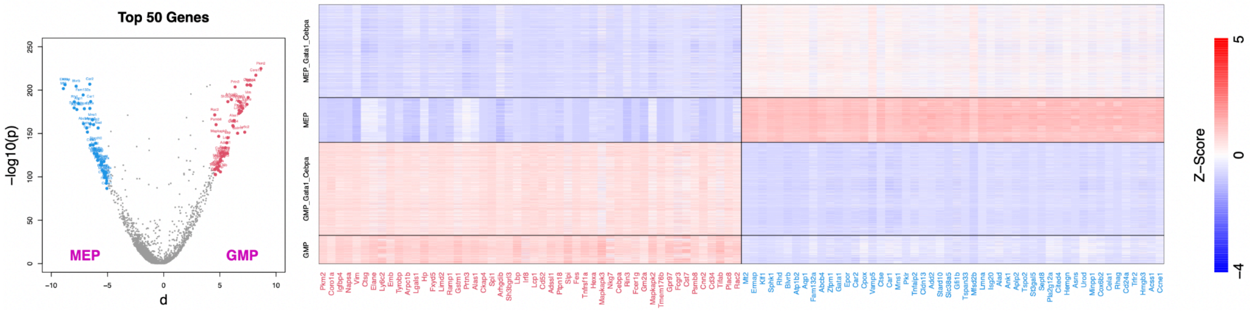

Top 50 Genes
−log10(p)
d
MEP
GMP
MEP_Gata1_Cebpa
MEP
GMP_Gata1_Cebpa
GMP
Z−Score
5
0
−4

Figure S6. Marker expression in *Gata1-Cebpa* module-induced transcriptomes. We examined whether key markers could be recapitulated by Manatee predictions. We defined GMP and MEP markers as the top 50 highly expressed genes. We highlighted such genes on the gene volcano plot. Markers for GMP and MEP were marked as red and blue, respectively. P-values were calculated using the two-sided U-test. D-values represent average gene expression differences. We further visualized the behavior of such markers within original GMP and MEP cells, as well as predicted GMP (GMP_P) and MEP (MEP_P) cells using the normalized expression heatmap. Gene-wise z-scores were used to quantify normalized expression profiles. We found the recapitulation of marker expression by Manatee.

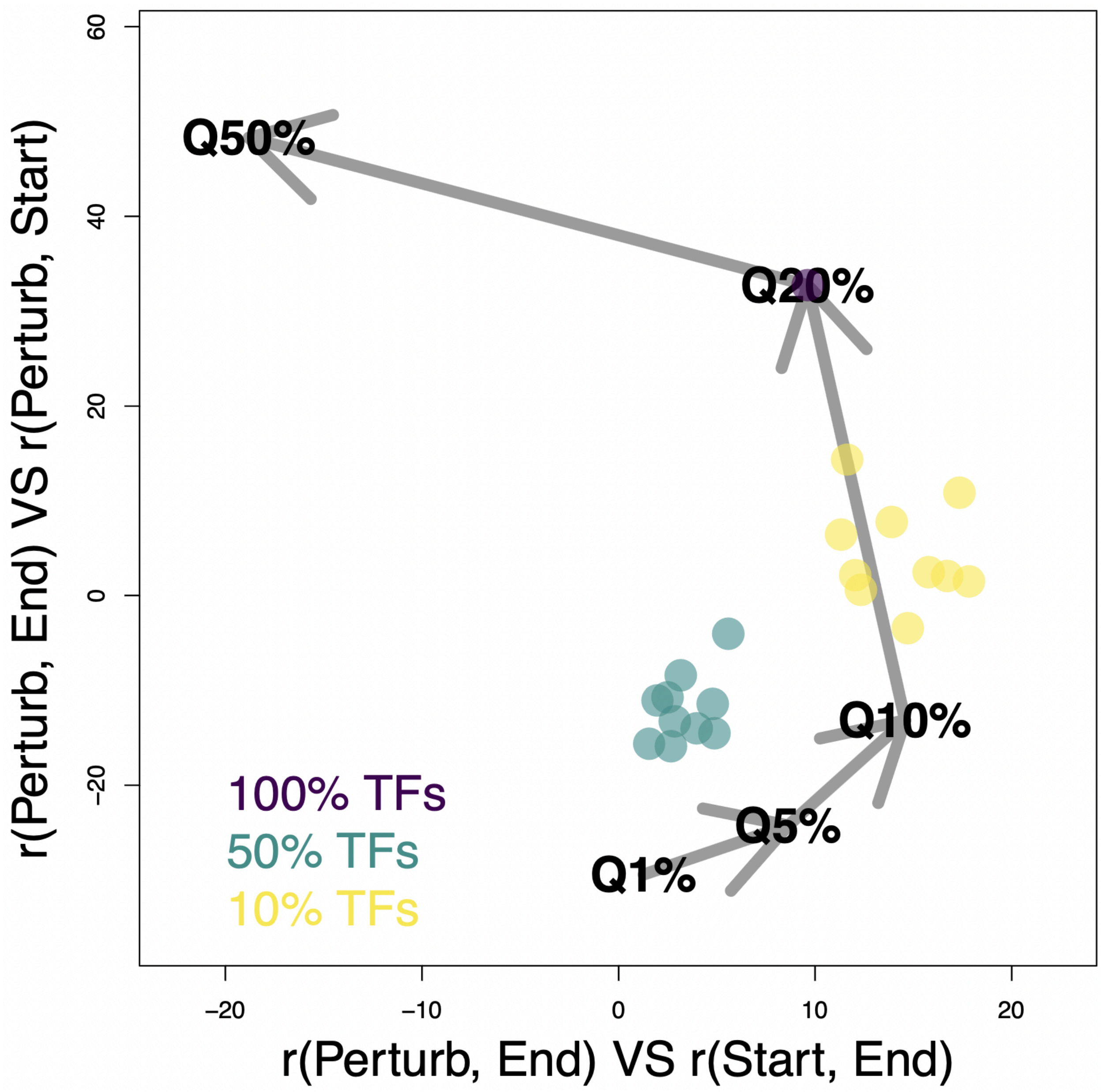

Q50%
Q20%
Q10%
Q5%
Q1%
100% TFs
50% TFs
10% TFs
60
40
20
0
−20
−20
−10
0
10
20
r(Perturb, End) VS r(Start, End)
r(Perturb, End) VS r(Perturb, Start)

Figure S7. Manatee analysis with different TF set sizes and latent value setups. We examined the performance of Manatee under various TF set sizes and latent value sampling quantiles. We evaluated the performance using the assessment plot. Q values examined were 1%, 5%, 10%, 20% and 50%. We analyzed the TOME lineage transition from “E10.5:Retinal primordium” to “E11.5:Retinal pigment cells”. Such a transition was “over-perturbed” (Figure S2) under the default Q (1%). We explained that such an “N.S.” perturbation is caused by overestimated latent values. Meanwhile, we observed that an underestimated latent space also deviates the Manatee prediction. A proper Q setup, in this case 5% solved the problem. Under the optimal Q, we further downsampled the TF set size to its 10% and 50%. We observed that reducing TF set size compromises the performance of Manatee.

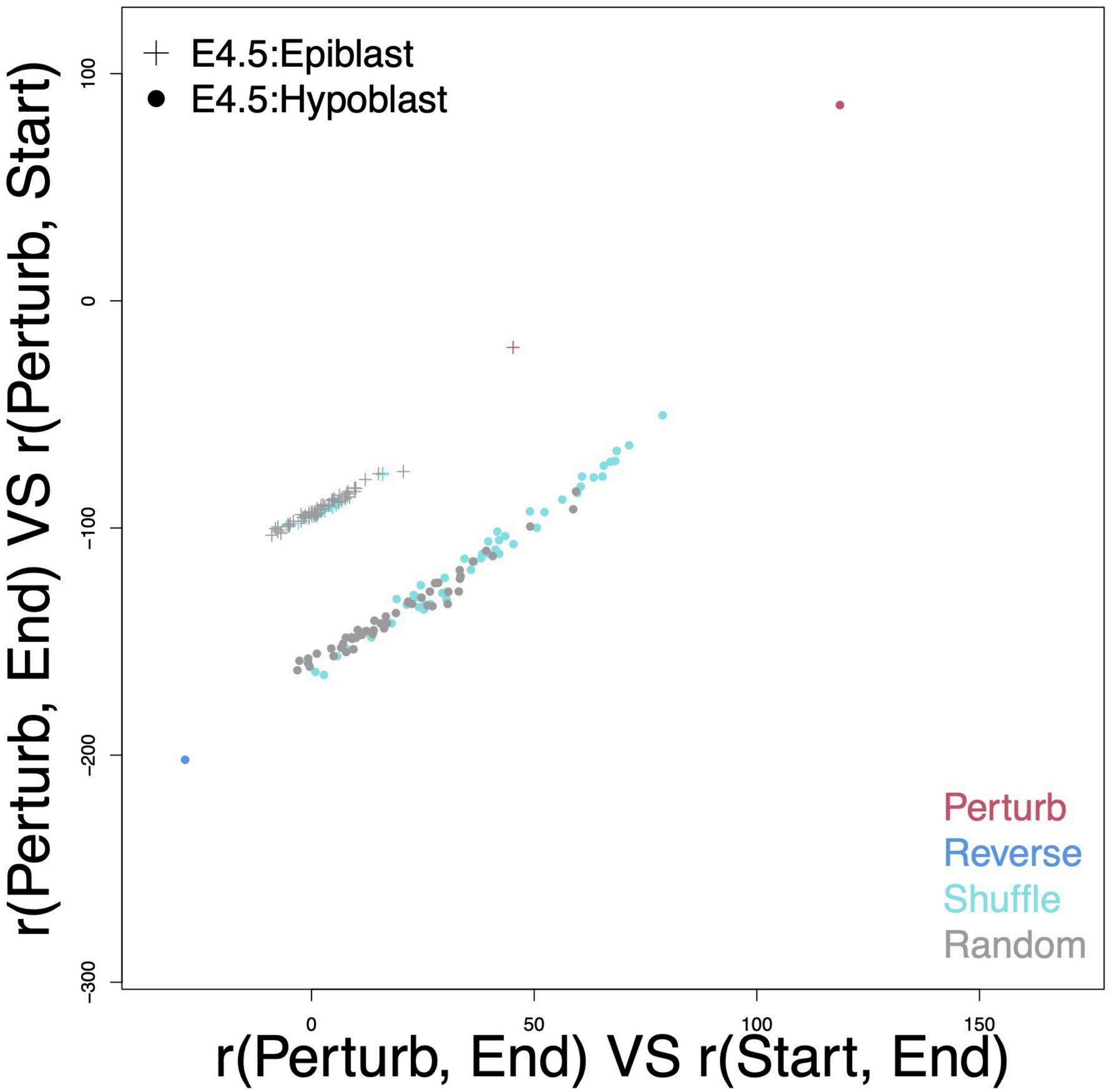
E4.5:Epiblast
E4.5:Hypoblast
Perturb
Reverse
Shuffle
Random
100
0
-100
-200
-300
0
50
100
150
r(Perturb, End) VS r(Perturb, Start)
r(Perturb, End) VS r(Start, End)

Figure S8. Manatee analysis with reversed, shuffled and random TF sets. We examined the performance of Manatee with several biologically irrelevant TF sets as the "null model". Perturb (red), the actual perturbation to be analyzed. Reverse (blue), the same set of TFs as "perturb" but with reversed directions. Shuffle (cyan), the same set of TFs as "Perturb" but with randomly permuted perturbation directions. A total of 50 "Shuffle" samples were analyzed. Random (gray), a random set of TFs with the total number of up and down-regulations kept the same as "Perturb". A total of 50 "Random" samples were analyzed. Without losing generality, we analyzed the inner cell mass to hypoblast (dot) and epiblast (cross) lineage bifurcation, as in Figure 1. We found that all "null model" groups yield compromised perturbations as shown on the assessment plot.
